# Crosstalk between HER2 and PD-1/PD-L1 in Breast Cancer: From Clinical Applications to Mathematical Models

**DOI:** 10.3390/cancers12030636

**Published:** 2020-03-10

**Authors:** Regina Padmanabhan, Hadeel Shafeeq Kheraldine, Nader Meskin, Semir Vranic, Ala-Eddin Al Moustafa

**Affiliations:** 1 Department of Electrical Engineering, Qatar University, 2713 Doha, Qatar; regina.ajith@qu.edu.qa; 2 Biomedical Research Centre, Qatar University, 2713 Doha, Qatar; hk1805332@student.qu.edu.qa; 3 College of Pharmacy, QU Health, Qatar University, 2713 Doha, Qatar; 4 College of Medicine, QU Health, Qatar University, 2713 Doha, Qatar; svranic@qu.edu.qa

**Keywords:** HER2, PD-1/PD-L1, HER2/PD-1 interaction, breast cancer, mathematical model

## Abstract

Breast cancer is one of the major causes of mortality in women worldwide. The most aggressive breast cancer subtypes are human epidermal growth factor receptor-positive (HER2^+^) and triple-negative breast cancers. Therapies targeting HER2 receptors have significantly improved HER2^+^ breast cancer patient outcomes. However, several recent studies have pointed out the deficiency of existing treatment protocols in combatting disease relapse and improving response rates to treatment. Overriding the inherent actions of the immune system to detect and annihilate cancer via the immune checkpoint pathways is one of the important hallmarks of cancer. Thus, restoration of these pathways by various means of immunomodulation has shown beneficial effects in the management of various types of cancers, including breast. We herein review the recent progress in the management of HER2^+^ breast cancer via HER2-targeted therapies, and its association with the programmed death receptor-1 (PD-1)/programmed death ligand-1 (PD-L1) axis. In order to link research in the areas of medicine and mathematics and point out specific opportunities for providing efficient theoretical analysis related to HER2^+^ breast cancer management, we also review mathematical models pertaining to the dynamics of HER2^+^ breast cancer and immune checkpoint inhibitors.

## 1. Introduction

The incidence of all types of cancers is increasing at an alarming rate, among which breast cancer (BC) is rated as the most common type (25% of all cancers) in many countries [1]. At the molecular level, there are four subtypes of BC: Luminal A, Luminal B, human epidermal growth factor receptor-positive (HER2^+^), and triple-negative (or basal-like) BCs [2,3,4]. These four main subtypes are categorized with respect to the presence of estrogen receptor (ER^+^), progesterone receptor (PR^+^), HER2, epidermal growth factor receptor (EGFR^+^), and basal markers such as cytokeratin 5/6. The subtype HER2 that constitutes 15–20% of all BC types is identified by the overexpression of HER2 receptor, which is associated with poor prognosis. 

HER2^+^ breast carcinomas are usually high-grade carcinomas (grade 3), associated with the comedocarcinoma phenotype and aggressive clinical behavior. Most of HER2^+^ breast carcinomas are ductal carcinomas, although some special types (e.g., Paget’s disease of the breast, apocrine carcinoma) may have a higher propensity for HER2 positivity [5,6,7]. Invasive carcinomas with HER2 positivity frequently arise from preinvasive lesions (ductal carcinoma in situ/DCIS), which frequently overexpress HER2. HER2 positivity is based on the amplification of the HER2 gene (HER2/chromosome enumeration probe 17 (CEP17) ratio ≥ 2.0) that causes complete, intense circumferential membranous expression of HER2 receptor in > 10% of cancer cells [8]. A subset of HER2^+^ breast carcinomas may also express steroid receptors: estrogen (ER) and progesterone (PR) receptors (Luminal B tumors). These tumors appear to be clinically less aggressive than HER2^+^ breast cancers and are associated with a better prognosis [9].

Treatment of HER2^+^ BC patients has undergone a significant improvement with the use of targeted therapeutic agents. For instance, Herceptin (trastuzumab) is the first FDA (The food and drug administration)-approved drug for HER2^+^ BC, and is the most commonly used drug for these patients. However, only 50–80% of patients with HER2^+^ BC benefit from this drug, while 20–50% either do not respond from the beginning of the treatment or develop resistance after treatment [10,11]. Moreover, with currently available treatment options, the overall disease-free survival (DFS) statistics of patients with the HER2^+^ subtype of BC are not adequate [12,13,14,15,16,17,18]. Hence, intense research is ongoing to determine new therapeutic targets or agents to improve the treatment of HER2^+^ BC patients. One of the areas showing encouraging results is immunotherapy. Novel immunotherapeutic strategies that target immune checkpoint pathways by altering T cell receptor (TCR) signaling have revolutionized treatment options for all types of cancers. Several studies show that immune therapy can boost the outcome of chemotherapy, radiotherapy, and/or other targeted therapies [19,20,21]. The immunogenicity of BC and the applicability of immunogenic pathways to enhance the treatment outcome of BC patients were not explored much in earlier days. However, in light of the motivating success rates of immunotherapy in other types of cancers, there is an increased interest in this area of research [16]. Out of all different subtypes of BC, TNBC and HER2^+^ BCs are identified to have a significant association with the immune surveillance of the host [19,22]. These studies also highlight the significance of immunomodulation, the number of tumor-infiltrating lymphocytes, and the expression of programmed death-ligand (PD-L1) to the disease prognosis and treatment outcome pertaining to human BC [16,22,23,24]. All these findings emphasize the importance of further investigating the applicability of immunotherapy for HER2^+^ BC. Out of the many possible ways to enhance immune surveillance and immune response, modulating immune checkpoint inhibitor pathways via the programmed death receptor-1 (PD-1)/programmed death ligand-1 (PD-L1) axis is one of the upcoming promising strategies to improve BC therapy [25]. 

**Figure 1:**
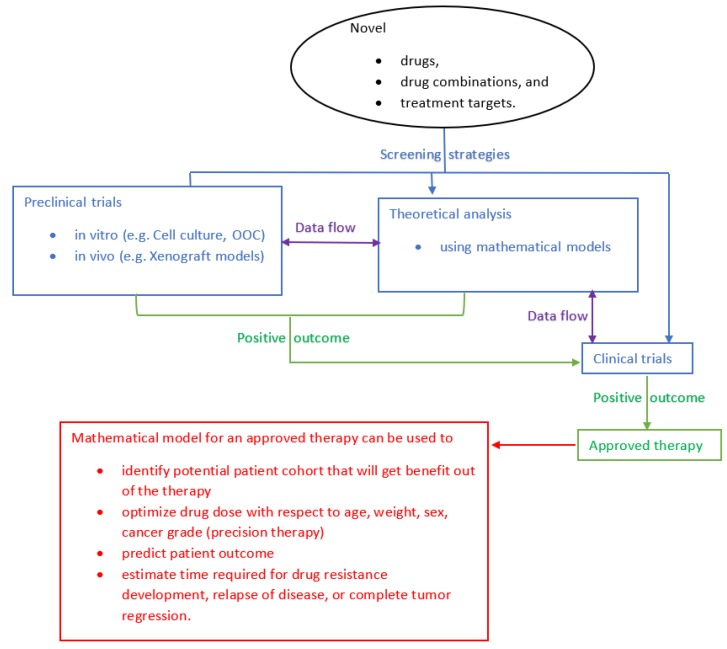
Schematic diagram showing the interdependence between preclinical trials, mathematical model-based analysis, and clinical trials at different levels involved in screening of new drugs, drug combinations, and drug targets and their use in optimizing patient outcome. OOC: organs-on-chips.

Meanwhile, another important area of research that is gaining popularity among mathematicians and engineers is the mathematical modeling of cancer dynamics and treatment. This increased interest is mainly due to the potential of mathematical models to contribute to the management of cancer. When it comes to the evaluation of new drugs, drug combinations, and drug targets, preclinical and clinical trials are imperative. As shown in Figure 1, along with preclinical and clinical trials, mathematical models are also used for drug screening before getting approval for clinical use and treatment planning [26,27,28,29,30,31,32,33,34]. The data flow arrows in Figure 1 indicate the use of experimental data from preclinical and clinical trials to devise mathematical models. On the other hand, the results obtained from theoretical analysis using mathematical models (e.g., tumor doubling time, optimal drug dose, predicted tumor volume, estimated time for relapse of disease) are used to optimize the clinical experiment and proposed therapeutic strategy [31,35,36,37,38]. Specifically, mathematical models can be used to analyze drug distribution (pharmacokinetics), drug response (pharmacodynamics) to monotherapy and combination therapy, development of drug resistance, and effect of drug toxicity related to cancer treatment [39,40]. Even though substantial efforts have been dedicated to the development of mathematical modeling of various types of cancers and their treatments, these contributions have been isolated from the clinical framework of cancer care and management. This has hindered the development of mathematical model-based innovative and translational treatment strategies that would otherwise benefit patients and clinicians in terms of easy and cost-effective treatment analysis and solutions. 

Mathematical models that are used to facilitate better therapeutic strategies are built using the knowledge of existing therapeutic agents and mechanisms involved in cancer initiation, progression, and treatment response. Hence, in Section 2 of this paper, we review the currently used therapeutic agents for HER2^+^ BC, highlighting the associated treatment response and development of drug resistance. Immune checkpoint inhibition is one of the proposed treatment strategies for HER2^+^ BC which is undergoing screening at different levels. Hence, in Section 3, we detail the significance of the PD-1/PD-L1 axis in HER2^+^ BC. In Section 2 and Section 3, along with the discussion on biological aspects of HER2 and the PD-L1 axis, we highlight how parallel theoretical analysis using mathematical modeling can contribute to improving the treatment of HER2^+^ BC. Next, in Section 4, we point out existing mathematical models related to the dynamics of HER2^+^ BC and immune checkpoint inhibitors; then, we provide a general mathematical model that can be used to develop related specific models, and finally, we list some of the research gaps in this area.

## 2. Current HER2^+^-Targeted Therapeutic Agents and Drug Resistance

**Figure 2:**
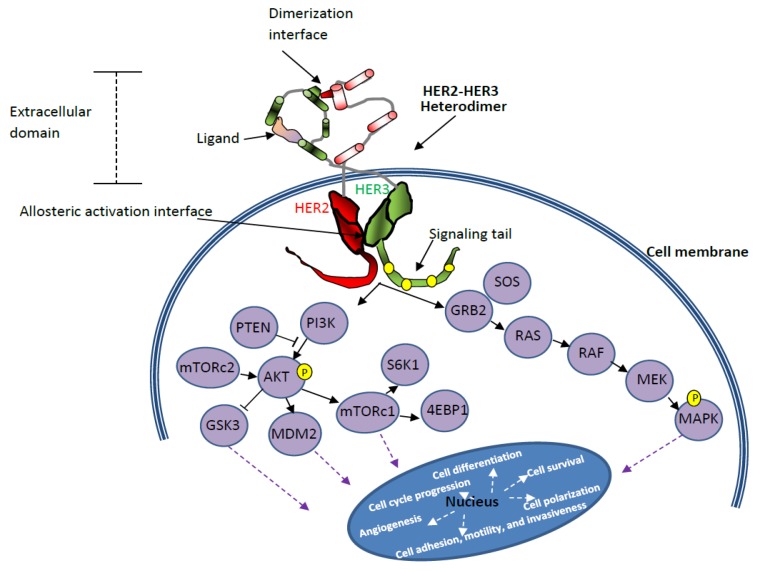
HER2–HER3 heterodimer and HER2 pathway. The main elements of this pathway are phosphoinositide-3-kinase (PI3K), phosphatase and tensin homolog (PTEN), protein kinase B homolog (AKT(PKB)), glycogen synthesis kinase (GSK), mouse double minute-2 homolog (MDM2), mammalian target of rapamycin complex-1,2 (mTORc1,2), ribosomal protein S6 kinase beta-1 (S6K1), 4E-binding protein-1 (4EBP1), growth factor receptor-bound protein-2 (GRB2), son of sevenless (SOS), retrovirus-associated DNA sequences (RAS), rapidly accelerated fibrosarcoma (RAF), MEK mitogen-activated protein kinase phosphorylates MAPK, P-phosphorylation, human epidermal growth factor receptor (HER) [44,45].

**Figure 3:**
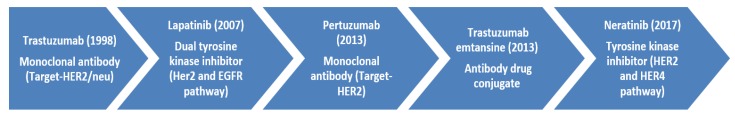
HER2-targeted therapeutic agents. Information in the chart includes the name of the drug, year of U.S. FDA approval, and type of receptor in order.

Targeted therapeutic agents used for the treatment of HER2^+^ BC mainly work by interrupting the tyrosine kinase-mediated downstream signaling by the HER2 receptor. Figure 2 shows that HER2 mediates the gene transcription pathway that regulates cell proliferation, differentiation, invasion, angiogenesis, metastasis, and cell survival. Due to the correlation of the HER2 pathway with different hallmarks of cancer, this pathway opens several potential therapeutic targets. Drugs such as trastuzumab (Herceptin) target the HER2 receptor and block growth signals of cancer cells. Other FDA-approved therapeutic agents that are currently in use for HER2^+^ BC include lapatinib (Tykerb), pertuzumab (Perjeta), ado-trastuzumab emtansine (Kadcyla), and neratinib (Nerlynx) (Figure 3). Trastuzumab is most effective in cancer cells with HER2 homodimers, and the drug recognizes the extracellular domain of the HER2 receptor [12,41,42,43]. 

HER2^+^ targeting drugs such as ado-trastuzumab emtansine (T-DM1) use trastuzumab as a drug-targeting agent to deliver emtansine to the HER2^+^ BC cells [46]. Thus, T-DM1 is a conjugate of a HER2-specific antibody (trastuzumab) and a cytotoxic drug, which is a derivative of maytansine (DM1). This antibody–drug conjugate (ADC) utilizes target specificity of trastuzumab to bind with HER2 and facilitate receptor-mediated internalization of T-DM1, followed by the release of cytotoxic DM1 via proteolytic digestion. T-DM1 thus inhibits HER2-mediated signal transduction and causes antibody-dependent cell-mediated cytotoxicity (ADCC) [46]. Compared to trastuzumab, pertuzumab blocks cancer cell growth with a similar mechanism but by attaching to a different part of the HER2 receptor. Pertuzumab prevents ligand-induced dimerization and subsequently inhibits downstream signaling. This drug is particularly effective against the most potent HER2-HER3 heterodimer. Notably, the combination of pertuzumab, trastuzumab, and docetaxel can substantially improve the treatment outcomes of HER2^+^ BC patients [16,47,48]. While targeted therapy agents work from outside the cell, small molecule agents such as lapatinib and neratinib, tyrosine kinase inhibitors, affect the chemical signals within the cancer cells. Both lapatinib and neratinib bind to ATP binding sites; however, when lapatinib binds reversibly, neratinib binds irreversibly [49,50]. Neratinib is usually used after the treatment with trastuzumab to reduce the recurrence of BC. Lapatinib blocks a protein that induces uncontrolled cell growth and is recommended for patients with trastuzumab-resistant BC [51]. 

Several mathematical models have been developed based on the experimental results pertaining to the biological aspects of HER2. For instance, in [52], the authors report a 3-compartmental cell-cycle model using the experimental data reported in [53] to depict the association of cell-cycle and overexpression of HER2 receptors. Specifically, HER2 overexpression is linked to shorter G_1_-phase and consequently early S-phase entry during cell cycle. Similarly, other types of biological information such as the number and type of antibody binding sites on HER2 receptors, target specificity of antibodies, and efficacy in releasing single or multiple drug conjugates to the site are important while quantifying the ADCC of an antibody–drug conjugate (ADC) [54,55]. These drug/receptor-specific details are used while devising related mathematical models as shown later in Section 4 of this paper. 

Treatment benefits of FDA-approved non-cleavable ADC (T-DM1) in HER2^+^ BC have motivated the development of different variants of ADC that can effectively release multiple cytotoxic agents (payloads or warheads) at the target. For instance, hertuzumab-based ADC (RC48) shows improved efficacy compared to trastuzumab, lapatinib, and T-DM1 in the resistant BT474/L1.9 xenograft model [42]. MEDI4276 (trastuzumab scFv with AZ13599185, a tubulysin payload), PF-06804103 (anti-Trop2 Aur0101), A166 (undisclosed payload), ALT-P7 (HM2-monomethyl auristatin E), ARX 788 (monoclonal antibody with monomethyl auristatin E), DHES0185A (monoclonal antibody with benzodiazepine monoamide), and SYD 985 (trastuzumab duocarmazine with seco-DUBA) are other ADCs under investigation for HER2^+^ BC [42,56,57,58,59,60,61,62]. With respect to the promising performance in Phase I and II clinical trials for the treatment of HER2^+^ metastatic BC, in December 2019, the FDA granted accelerated approval for trastuzumab deruxtecan (DS-8201) [12,63,64,65]. When it comes to the desired properties of drugs, it is important to have optimal stability properties while the drug moves through the human plasma, along with efficient target-specific drug release [42,56,57,58,59,60,61,62]. Out of the above mentioned ADCs, preclinical experiments on animal models are reported only for MEDI4276, RC48, ARX 788, DS-8201, and SYD 985, and hence more in vitro and in vivo experiments are imperative in this area [59,61,62,66]. Similarly, mathematical models that depict the dynamics of these novel drugs, as well as many of FDA- approved anti-HER2 agents are yet to be devised. For instance, in [67], the authors discuss a mathematical model-based analysis to determine the optimal drug dose and treatment plan for the use of lapatinib as a treatment for glioblastoma. A similar theoretical analysis can be done for the use of anti-HER2 drugs for HER2^+^ BC. Even though there are mathematical models related to the use of trastuzumab and T-DM1 for HER2^+^ BC [55,68,69,70,71], similar models related to lapatinib, pertuzumab, and neratinib are yet to be reported. 

The long list of novel drugs that are under investigation for the treatment of HER2^+^ BC invokes hope. However, the development of drug resistance is a common event that often curtails the long-term use of many therapeutic agents and thus squanders the effort and money spent on bringing these novel drugs from bench-to-bedside [72,73,74,75,76,77,78]. Ineffective or impaired binding of drugs to HER2 receptors, switching of signaling pathways, and metabolic reprogramming are some of the common factors that retain the characteristics (abnormal proliferation and anti-apoptotic) of the disease [15,79]. For instance, even though the drug resistance mechanism of trastuzumab is not completely understood, the activation of phosphatidylinositol 3-kinase (PI3K) signal transduction pathway is considered as one of the key mechanisms of resistance. The PI3K–AKT pathway promotes the growth and survival of cells via extracellular signals (Figure 2). Increased PI3K/AKT phosphorylation and signaling were linked to blocking trastuzumab effects on HER2-overexpressing breast tumors [80]. This blockage is mediated by the decreased levels of the phosphatase and tensin homolog (PTEN), which is strongly related to a much poorer response to trastuzumab [80]. In vitro and in vivo studies suggest that reversing this effect by using PI3K inhibitors (e.g., alpelisib, copanlisib) or mammalian target of rapamycin (mTOR) inhibitors (e.g., everolimus), helped to overcome trastuzumab resistance [15]. Similarly, the overexpression of membrane-associated glycoprotein mucin 4 (MUC4) and increased insulin-like growth factor-I receptor (IGF-IR) signaling were found to be more common among cells that are resistant to trastuzumab. 

Around 25–30% of the HER2^+^ BCs express an abnormal form (lacking the extracellular domain) of the HER2 fragment known as p95HER2, along with the normal HER2 receptor [81,82]. The presence of p95HER2 can make HER2^+^ cancer cells resistant to drugs such as trastuzumab as the monoclonal antibody cannot detect aberrant p95HER2 receptors. However, cells with p95HER2 receptor respond to tyrosine kinase inhibitors such as lapatinib [82]. Another factor that induces resistance to trastuzumab is the lack of CD16A (cluster of differentiation) receptor or inefficient binding with the CD16A receptor. CD16A (FcγRIII) is found on immune cells, and many studies have demonstrated the role of CD16A in inducing ADCC. Overexpression of neuromedin U (NmU) is related to the expression of TGF-β (Transforming growth factor) and PD-L1 in the tumor microenvironment, which in turn is associated with impaired ADCC. Hence, the use of immune checkpoint inhibitors in NmU-overexpressing tumors may revert or prevent resistance to trastuzumab [15,83,84]. In short, trastuzumab resistance is mediated by (1) impaired interaction of trastuzumab to HER2 (via MUC4, p95HER2, CDK2), (2) an altered or parallel intracellular PI3K/AKT/mTOR signaling pathway, (3) mutation of PIK3CA gene, and (4) higher levels of cyclin-E, fatty acid synthase (FASN), and/or NmU. Similarly, potential reasons for T-DM1 resistance include difficulties in binding with the receptor (due to MUC4, p95HER2), impaired receptor internalization, improper release of cytotoxic agent, and/or activation of parallel pathways [85,86]. All of the FDA-approved anti-HER2 drugs are associated with resistance development via one or more of the above-listed pathways. Compared to trastuzumab, lapatinib and neratinib bind to the intracellular domain of the HER2 receptor and apparently, the extracellular domain-mediated drug resistance pathways are ineffective for these drugs. However, these drugs are associated with primary as well as acquired drug resistance-mediated treatment issues [15,87,88]. All of these studies show clearly that HER2 is an important target for HER2^+^ BC; however, resistance to different types of HER2 drugs is still a major issue in the management of human cancers expressing HER2 including breast. Thus, the potency of new targets such as the PD-1/PD-L1 axis that can be used in combination with anti-HER2 drugs needs to be investigated further.

Several mathematical models have been used to study the response (e.g., drug sensitivity, inherent drug resistance, drug-induced resistance) of the heterogeneous tumor microenvironment to various therapeutic interventions such as chemotherapy [89], radiotherapy [90], and hormone therapy [91], in general [92]. However, mathematical models that analyze drug resistance development pertaining to anti-HER2 therapy in particular are scarce. For example, in [93], the levels of prostate-specific antigen (PSA) were used to predict the development of castrate resistance in prostate cancer cells and to compare the efficacy of intermittent and continuous androgen deprivation therapy. Similar models can be used in the case of HER2^+^ BC, specifically by modeling the presence of impaired receptors and overexpression of certain proteins, peptides, or cytokines (e.g., MUC4, NmU, TGF-β) which can give quantitative insights into the mechanism related to drug resistance development. Such models can help optimize treatment schedules and determine effective drug combination so as to curtail drug resistance development.

## 3. PD-1/PD-L1 and HER2 Crosstalk in Breast Cancer

Immune checkpoint inhibition is an intensively investigated but yet-to-be approved therapeutic strategies for HER2^+^ BC patients. Hence, in this section, we first introduce the PD-1/PD-L1 axis and then point out: (1) the level of PD-L1 expression in HER2^+^ BC, (2) the association of PD-L1 expression to disease progression and response to therapy, and (3) some of the factors that are linked to the overexpression of PD-L1. As mentioned in Section 2, these biological aspects are needed while developing a mathematical model to represent the treatment scenario facilitated by a single agent (e.g., anti-HER2 alone) or multiple agents (e.g., anti-HER2 with immune checkpoint inhibition) for HER2^+^ BC. 

**Figure 4:**
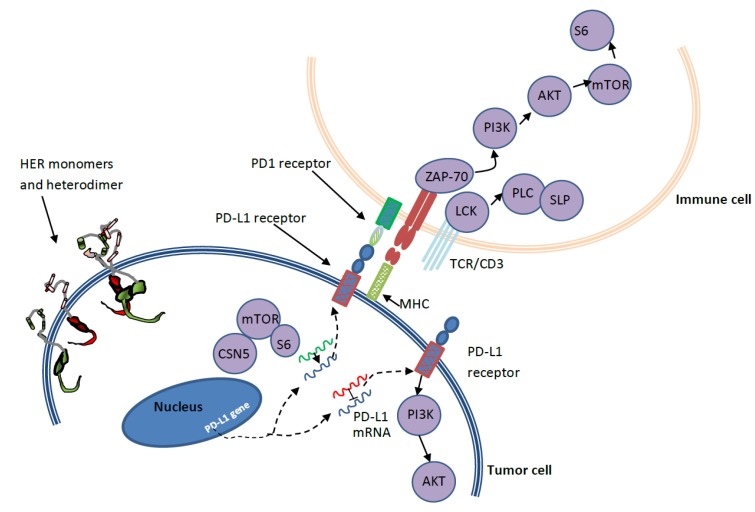
Upregulation of programmed death ligand-1 (PD-L1) and immune evasion by a HER^+^ tumor cell [45,95]. MHC: major histocompatibility complex, ZAP-70: zeta-chain-associated protein kinase-70, PLC: phospholipase C, SLP: SH2-leukocyte protein, LCK: lymphocyte-specific tyrosine kinase, CSN5-Cop9: signalosome complex, PD-1: programmed death receptor-1.

Several co-inhibitory and co-stimulatory pathways that are regulated by the immune system mediate the selective attack on external invaders (pathogens) while sparing the host cells [94]. The human body has innate and adaptive immune mechanisms in place to facilitate immune response depending on the type of pathogens. The programmed death receptor-1 (PD-1/CD 279) and its ligand programmed death ligand-1 (PD-L1/ B7-DC) are involved in one such mechanism that exists to avoid autoimmunity (attack on host cells). The PD-L1 on host (normal) cells interacts with the PD-1 receptor on immune cells to avoid an attack (Figure 4). The same PD-1/PD-L1 pathway is utilized by cancer cells to evade immune attack. Hence, when the PD-1 receptor on immune cells interacts with the PD-L1 on cancer cells, immune response activities such as T cell activation and T cell proliferation are halted. In some cancers, even if cancer cells are immunogenic, they are also identified to have many receptors (e.g., PD-L1, PD-L2) to stimulate immune checkpoint targets (e.g., PD-1) and block the immune response. Other immune checkpoint targets include cytotoxic T lymphocyte antigen (CTLA-4), glucocorticoid-induced TNFR-related protein (GITR), OX40, 4-1BB, T-cell immunoglobulin (TIM-3), and lymphocyte-activation gene (LAG-3) [16]. 

Immune checkpoint inhibitors facilitate tumor cell lysis by reactivating immunologic actions, which were earlier blocked by tumor cells via immune checkpoints. Hence, many drugs (e.g., monoclonal antibodies, immunoglobulins, and small molecule inhibitors) that can facilitate immune checkpoint inhibition are undergoing clinical trials (e.g., NCT03523572, NCT03125928, NCT03523572) for the treatment of HER2^+^ BC [96]. Examples of anti-PD-1 monoclonal antibodies are pembrolizumab (Keytruda), nivolumab (Opdivo), and cemilimab (Libtayo). The most commonly used anti-PD-L1 monoclonal antibodies are atezolizumab (Tecentriq), avelumab (Bavencio), and durvalumab (Imfinizi) [97,98,99]. 

For the purpose of mathematical modeling, quantitative information regarding the level of PD-L1 expression, the association of PD-L1 overexpression and HER2 positivity in relation with various biomarkers, and their association with disease prognosis and treatment response has to be collected. Such a comprehensive base of quantitative data is essential to develop mathematical models that can identify the patient population that will benefit from targeting HER2 or/and PD-L1 axis for therapy. In this regard, we reviewed immunohistochemistry studies that are conducted to investigate the influence of PD-L1 expression in human BC. Such studies pointed out the significant influence of patient age, tumor grade, tumor type, and lymph node status on the expression of PD-L1. Moreover, the expression of Ki-67 and the absence of ER also show a significant influence [22]. In two studies that included all different molecular subtypes of BC, one study reported that PD-L1 was expressed in 152 (23.38%) specimens out of the 650, and the other reported 21.1% (89/870) [22,100]. The expression of PD-1/PD-L1 varied with stage and molecular subtypes of BC out of which TNBC has the highest expression followed by HER2^+^ subtype [22,100,101,102,103]. Noticeably, studies report a significant difference in the expression of PD-L1 in tumor (cell membrane (64%), cytoplasm (80%) and stromal (93%)) cellular compartments [104,105]. 

In the case of HER2^+^ BC, PD-L1 expression of up to 58%, 53.8%, and 32% is reported in tumor cells, immune cells, and both cells, respectively [106,107,108]. Up to 30.7% of PD-1 expression on tumor-infiltrating lymphocytes (TILs) around HER2^+^ BC is also seen. PD-1/PD-L1 expression in metastatic tumors was correlated with poor prognosis, whereas no relation to clinicopathological features was noted in primary tumors [109]. In essence, it can be seen from literature that high PD-L1 expression combined with an increase in T regulatory cells (Tregs) and a decrease of TILs are associated with poor survival [22,102,107,110]. However, high PD-1/PD-L1 expression along with a higher number of TILs in the tumor microenvironment is associated with improved OS (overall survival) and/or DFS (disease free survival) [24,111,112]. Similarly, PTEN expression is related to improved OS [102]. 

Several studies evaluate the importance of PD-1/PD-L1 expression on tumor cells and/or immune cells and the presence of TILs and Tregs in the tumor microenvironment in predicting response to treatment pertaining to BC [3,104,108,113,114]. Specifically, blocking immune evasion and attracting more TILs and fewer Tregs (reactivating immunogenicity of BC) to the tumor microenvironment can improve the OS of BC patients [19,23,115,116,117,118,119]. Sixteen percent of HER2^+^ subtypes are lymphocyte predominant (> 50–60% of TILs present) and are associated with improved outcome (EFS: event-free survival, OS) with many treatment modalities [19,120,121,122,123,124]. The FinHER trial demonstrated the link between improved response to trastuzumab and higher levels of TILs among HER2^+^ BC patients [125]. It showed that in the case of HER2^+^ tumors which are highly proliferative, the presence of TILs in the tumor microenvironment is a predictive biomarker for favorable responses to trastuzumab treatment [102,110,126,127]. As mentioned in [102,117,127] the experimental analysis on HER2^+^ patients suggests that CD8^+^ T cell-mediated cytotoxicity and PD-L1 expression together may predict improved outcome in HER2^+^ BC patients under combined chemotherapy and HER2-targeted therapy [102]. These findings emphasize the importance of tumor–immune interaction in BC progression [46,73,114,128,129]. Moreover, based on 6 different studies, in [130], the authors highlight that PD-L1 expression in both tumor cells and immune cells of the host can contribute to the overall response to treatment. Hence, the evaluation of an overall expression in both these cells is recommended as a predictive biomarker [23,115,116,117,118,119]. 

As per available literature, potential predictive biomarkers that can be used to select patients who may benefit from combined treatment using HER2-targeted and PD-1/PD-L1 axis based therapeutic agents are (1) HER2 amplification/overexpression, (2) PD-1/PD-L1 expression, (3) presence of a greater number of TILs and fewer Tregs, (4) higher TMB (tumor mutation burden), (5) PTEN expression, and (6) expression of CD5, CD74, CD96, and CD226, to name only few [38,86,121,131,132,133,134,135,136,137,138]. However, it is still not clear which combination of clinicopathological factors are most reliable predictive biomarkers to implement effective treatment protocols using anti-HER2 and/or PD-1/PD-L1 pathways [39,139]. Moreover, since blocking immune checkpoints can have side effects such as organ damage, careful analysis using multiple biomarkers is required while developing combination therapy protocols. Moreover, most of the in vivo and in vitro studies on the use of immune checkpoint inhibition are done using antibody-based drugs that are associated with the high cost and long half-life [140]. The efficacy of small molecule PD-1/PD-L1 inhibitors in implementing a combination of immunotherapy and HER2-targeted therapy to enhance the potency of BC treatment should also be investigated [141,142,143,144]. Along with the suggested experimental analysis, mathematical model-based analysis is also desired in this area. Similar to the immune checkpoint inhibition-based mathematical model given in [145], quantitative information about the association of PD-L1 expression, Tregs, and TILs in the tumor microenvironment can predict whether a HER2^+^ BC patient will benefit from immune checkpoint inhibition [115,127].

Steered by the association of HER2 and PD-L1 axis in BC dynamics, several new drugs and drug combinations (PD-1/PD-L1 and HER2-targeted) are under clinical investigation for the treatment of HER2^+^ BC. Specifically, HER2-targeted drugs such as trastuzumab/pertuzumab/T-DM1/pyrotinib/tucatinib/zenocutuzumab/margetuximab are used in combination with immunotherapeutic drugs such as nivolumab/durvalumab/atezolizumab/pembrolizumab/avelumab and other chemotherapy drugs. Currently, atezolizumab is the only FDA-approved immune checkpoint inhibitor for BC (TNBC) treatment. Out of many new drugs that are under clinical trial, margetuximab (MGA-H22) received FDA approval for fast track investigation on its potency to treat HER2^+^ metastatic BC [146,147,148]. This is a novel HER2-targeted monoclonal antibody tailored to enhance the binding affinity to multiple sites by mediating activation of Fc-γ receptors. Margetuximab showed improved ADCC compared to trastuzumab [131,147,149]. Hence, optimizing the functionality of Fc receptors to enhance ADCC is also a promising direction for improving the treatment of HER2^+^ BC [38,131,132]. 

As mentioned earlier, mathematical models that depict the pharmacokinetics and pharmacodynamics of these potential drugs, their combinations, and their effect on individual cell dynamics in the heterogeneous tumor microenvironment can accelerate the search for better treatment options for HER2^+^ BC (See Figure 1). Based on the knowledge of various HER2 signaling pathways (Figure 2), several HER2-targeted treatment options (Figure 3) are currently in use. However, even though PD-1/PD-L1 pathways are well discussed in literature, the exact mechanisms that lead to the overexpression of PD-L1 and their consequences in HER2^+^ BC patients are yet to be clearly understood [102,115,121,134,150,151]. Moreover, PD-1/PD-L1 signaling pathway-based therapeutic targets and agents for HER2^+^ BC patients are still under investigation. Available literature suggests that exposure to cytokines (interferon-gamma (IFN-γ), interleukin-4 (IL-4), granulocyte-macrophage colony-stimulating factor (GM-CSF), abnormalities in EGFR signaling, and genetic alterations (e.g., PIK3CA mutation) can induce PD-L1 overexpression [95,102,150,152]. The complex regulatory signaling pathways related to PD-L1 activation involve PI3K/PTEN/AKT/mTOR and retrovirus-associated DNA sequences (RAS)/rapidly accelerated fibrosarcoma (RAF)/MEK/mitogen-activated protein kinase (MAPK)-extracellular signal-regulated kinase (ERK), which are linked with transcriptional factors such as STAT1 (signal transducer and activator of transcription), STAT3, HIFs (hypoxia-inducible factors), c-Jun, and NF-κB. These factors can alter intercellular signaling and cell-cycle control [95,152]. Regulation of PD-L1 expression is also facilitated by mRNAs via translational enhancement/suppression [95]. 

**Figure 5:**
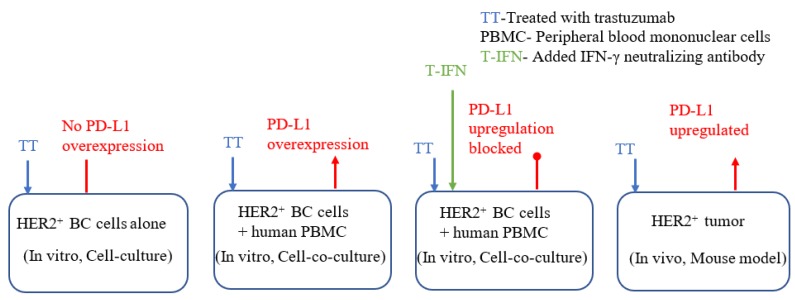
Expression of PD-L1 in different in vitro and in vivo settings [134]. IFN: interferon-gamma, BC: breast cancer.

A study that evaluated the involvement of the PD-L1 pathway in the development of drug resistance against an anti-HER2 drug revealed significant crosstalk between HER2 and PD-L1 pathways [134]. Specifically, IFN-γ is linked to the upregulation of major-histocompatibility complex (MHC-1) and PD-L1 in HER2^+^ BC cells. The inhibition of HER2 signaling (via gene knockdown or kinase inhibition) influenced PD-L1 expression in different ways for various settings, as shown in Figure 5. Specifically, in vitro experiment using trastuzumab on HER2^+^ BC cells showed no PD-L1 overexpression; however, when the same cells were co-cultured with human peripheral blood mononuclear cells (PBMCs), PD-L1 overexpression was noted. It can be seen that when IFN-γ was neutralized in the co-culture setup, PD-L1 upregulation was blocked. Similar to a trastuzumab-treated co-culture setup without anti-IFN-γ antibody, in the case of in vivo mouse model, PD-L1 was upregulated. Since the process of trastuzumab mediated ADCC involves engaging cancer cells with immune cells leading to the secretion of IFN-γ, the resulting overexpression of PD-L1 is postulated as a possible pathway of drug resistance development against trastuzumab treatment. Consistent with this finding is the synergistic response reported in a preclinical study using mice that tested human PD-L1 and HER2 gene vaccinations in treatment of HER2^+^ cancers [153]. In essence, there is evidence of crosstalk between HER2 and PD-L1 pathways; however, there is much more to know regarding the underlying interactions pertaining to these two pathways [150]. This calls for more research in this area which will be beneficial towards devising better treatment options for HER2^+^ BC.

## 4. Mathematical Models Used for Breast Cancer Management

In Section 2 and Section 3, along with the biological aspects pertaining to HER2 and PD-1/PD-L1 axis, we have pointed out the opportunities to improve HER2^+^ BC treatment using mathematical modeling. In this section, we review existing mathematical models in this area and detail a general one that can be used to devise novel models in the context of anti-HER2 therapy and/or immune checkpoint inhibition. It is apparent that mathematical models of tumor–immune interaction with respect to HER2-targeted therapy and/or immune checkpoint blockade can be used to explore tumor dynamics in detail and to answer questions that are difficult to answer by clinical analysis [54,71,145]. Since 1954, mathematical model-based analysis has contributed heavily to various areas of cancer research such as drug scheduling, estimating drug response in terms of desired effect, testing research hypothesis, and to study interdependence and sensitivity of various parameters involved in cancer dynamics [39,40,70,154,155,156,157]. At this point in time, it is worth noting that nuclear physics, neuroscience, epidemiology, and physical chemistry are fundamental areas of research that witnessed a big leap forward due to the integration of empirical and theoretical works. Unfortunately, even though extensive clinical investigations (empirical) and mathematical (theoretical) analysis were conducted in the area of cancer research, both areas stand as separate entities pursuing parallel paths. It is imperative to merge or interlace these two strategic areas of research to foster translational technologies that can revolutionize the area of medicine and biology (Figure 1).

**Figure 6:**
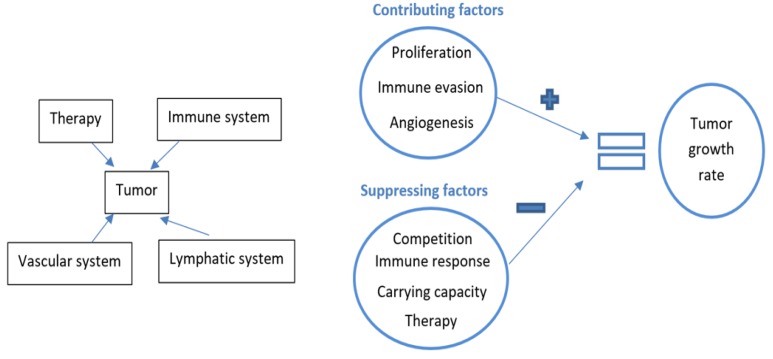
Factors influencing tumor dynamics.

In general, mathematical models are simplified quantitative representations of the complex and nonlinear phenomenon involved in cancer progression and regression. For instance, as shown in Figure 6, it is apparent that the overall growth rate of a tumor is influenced by the cumulative actions of the immune system, lymphatic system, vascular system, and the treatment used. The net tumor volume is due to the sum of all contributing factors minus all of the suppressing factors including the effect of treatment. Thus, such models can be used to investigate the efficacy of novel treatment strategies, and to find the optimal drug dose required for achieving certain desired drug response without actually testing on patients. Specific mathematical models of tumor–immune interaction with respect to the adopted mode of treatment such as chemotherapy [158], radiotherapy [159], immunotherapy [160], hormone therapy [161], anti-angiogenesis [162], nanomedicine-based cancer therapy [33], gene therapy and/or oncovirotherapy [163], and combination therapies [164] have been widely discussed recently. Many recent reviews summarize the history of mathematical models in various treatment areas related to cancer [33,40,156,165,166,167,168]. As mentioned earlier, one of the focusses of this paper is to highlight the progress achieved in the mathematical modeling of BC with specific emphasis on anti-HER2 treatments and immune checkpoint inhibitors. 

First of all, both in vivo and in vitro models were used to derive mathematical models of breast cancer (Figure 1) [1,26,50,55,155]. For instance, in [1], experimental evidence on the growth of the MCF-7 cell line, intercellular interaction between tumor cells, and interaction with the immune cells (natural killer (NK) cells, cytotoxic T-lymphocytes (CTLs), white blood cells (WBCs)) and estradiol (estrogen) was used to develop a mathematical model. Using the model, the authors demonstrate the existence of stable (dormant) microscopic tumors and their control or eradication by the immune system. In another study, heterogeneous cell lines (MDA-MB-468, SUM149PT, MDA-MB-231, and MDA-MB-453) were used to quantify the cellular uptake and treatment response to doxorubicin related to TNBC [26]. In vitro experiments used to develop mathematical models of BC are reported in [1,26,55]. Compared to in vitro models which are based on certain BC cell lines, experimental mice models (in vivo) are used more frequently to estimate parameter values of mathematical models [71,155]. Even though many experimental studies have used drosophila, zebra fish, and chicken embryo models for cancer research, mathematical models based on these animal models are scarce [169,170,171].

Next, as mentioned in the first part of this review paper, there are different types of investigational drugs (e.g., antibodies, inhibitors, ADC) and plausible drug combinations under consideration for improving treatment options for HER2^+^ patients. However, due to ethical and financial reasons, it is difficult to conduct clinical trials to evaluate all possible combinations [13,71,172]. Mathematical models can contribute to derive useful information in this regard. In several studies [1,71,173,174], the authors analyzed the tumor–immune interaction in BC with respect to the use of various drugs. Specifically, the three main mechanisms involved in tumor–immune interaction: (1) elimination of tumor by the immune system, (2) equilibrium status (stable, dormant) attained by the tumor under the action of the immune system, and (3) escape of the tumor from immune action leading to uncontrolled growth, were studied in detail using mathematical models [1,175,176]. Apart from depicting tumor–immune interactions, mathematical models were used to determine the growth of tumor in different stages of primary BC (T1a, T1b, T1c, T2, T3) in patients with no metastasis (M0) and no lymph node involvement (N0) [177]. Using the mathematical model, the authors were able to determine the critical diameter (2 mm) of tumor that the immune system can eliminate. They also discussed the annihilation of strongly antigenic and weakly antigenic BC tumors by the cytotoxic T-lymphocytes (CTLs) and macrophages, respectively. Another interesting study used a histological data-based mathematical model from a clinical trial (48 patients) to predict the response of BC patients to neoadjuvant chemotherapy [37]. In this study, the authors showed that it is possible to evaluate the treatment outcome of patients using the analysis of the parameters pertaining to vasculature development-based biomarkers in primary BC.

In the case of combination therapies, apart from predicting effective drug combinations, mathematical models can be used to determine effective order of drug administration. In [55,178], the authors investigated the influence of the order of drug administration on treatment outcomes when multiple drugs are used to treat BC. Some mathematical models specifically portray mechanisms related to the HER2^+^ BC subtype [55,70,71]. In one study, experimental and computational analysis was conducted to evaluate the efficacy of using trastuzumab along with paclitaxel and the influence, if any, of the order of drug administration in treatment efficacy [55]. Using a HER2^+^ cell line (BT474), the authors found that there is more synergistic interaction between the drugs when trastuzumab is administered first and then paclitaxel. In [71], the authors described a mathematical model of tumor–immune interaction with respect to trastuzumab treatment. This model predicted increased infiltration of leukocytes in treated animals. This conclusion was validated using a parallel experimental analysis (Section 3). This study emphasizes the power of mathematical models to give quantitative results pertaining to cancer dynamics and the effect of treatment. 

Apart from evaluating treatment efficacy, mathematical models can be used to test hypotheses related to the mechanism of tumor–immune–drug interactions. For instance, in [70] the authors explored trastuzumab mediated internalization of receptors (in SKBR3 cells) associated with the HER2 pathway to determine why HER2 is resistant to down-regulation [179]. Using the mathematical model developed, the authors pointed out the mismatch in the experimental data with the hypothesis of the fast recycling of HER2 receptor back to the plasma membrane. However, the mathematical model described the sustained internalization of receptors in cells with ruffles in the membrane. Moreover, the model predicted that the receptor internalization occurs three times slower for non-ruffled cells than ruffled cells.

As in the case of many types of cancers, in the case of HER2^+^ BC, poor response ratio to treatment as well as the development of drug resistance, are linked to the presence of BC stem cells (BCSCs). This is mainly due to the fact that the treatment often can eradicate only differentiated cancer cells but not cancer stem cells (CSCs). Mathematical model-based analysis can also be used to comment on the possibility of tumor initiation within a clinically observable time with respect to the presence of BCSCs [28]. Here, we note that the high levels of PD-1/PD-L1 expression reported on CSCs are believed to aid immune evasion [180,181]. The expression of PD-L1 in BCSC is linked to enhancing the presence of transcription factors such as OCT-4A and Nanog [180]. This enhanced expression of transcriptional factors is believed to retain the stem cell nature of BCSC via the PI3/AKT pathway. Such associations of BCSC and PD-1/PD-L1 expression in HER2^+^ BC are yet to be represented using mathematical models towards gaining further insight into their underlying mechanisms.

Looking at the immune checkpoint pathway, since it is a recent addition to the treatment strategies for cancer, the mathematical modeling attempts in this area are relatively less compared to other treatment modalities [54,145]. The first mathematical model depicting the dynamics of tumor microenvironment concerning anti-PD-1 treatment was reported recently in [145]. Specifically, a mathematical model for combination therapy using a vaccine and an immune checkpoint inhibitor is presented. Since these two drugs complement each other, the mathematical model is used to evaluate the synergistic action of these drugs and propose the use of synergy maps to determine drug dose that respects the constraints such as maximum tolerated dose (MTD). The authors used 13 partial differential equations to illustrate the dynamics involved in cancer cells, immune cells (CD4+ and CD8+), cytokines (IL-2, IL-12), vaccine-induced colony-stimulating factor (GM-CSF), and inhibitor associated with PD-1. In [54], the authors discussed the mathematical modeling of co-culture (in vitro) of natural killer (NK) cells, cancer cells, and antibody to study the anti-body dependent cell lysis. In [182], the authors revalidated the findings in [145] using experimental data from mice models. Based on the analysis concerning tumor-free and tumorous equilibria, the authors recommend the use of combination therapy since immune checkpoint inhibition alone is not sufficient to maintain a tumor-free equilibrium. This conclusion is consistent with the results of experimental and clinical studies in this area. A good review of the pharmacokinetics (PK) and pharmacodynamics (PD) of the first five FDA-approved drugs that come under the heading of clinical checkpoint inhibitors is discussed in [39].

The mathematical models discussed in [39,54,145,182], in relation to immune checkpoint pathways are general and not for BC in particular. In 2019, the first in silico trial with the use of immune checkpoint inhibitors (anti-CDLA-4, anti-PD-L1) in patients with metastatic BC has been reported [175]. In this paper, mathematical models are used to explain immune suppression and evasion in tumor-draining lymph node and tumor microenvironment. This model also accounts for PD-L1 expression, the intensity of antigen, effects on the immune system, and response to checkpoint inhibition. The authors claim that with sufficient clinical measurements, this model can predict the treatment response of individual patients concerning various treatment modalities. 

Another promising experimental model that can be used to contribute to both empirical, as well as theoretical investigations pertaining to HER2^+^ BC, is organs-on-chips (OOC). OOC are a novel in vitro platform for aiding the development and testing of therapeutic drugs for cancer [183,184]. Since OOC incorporate multiple cellular and biophysical functional features together, they can recreate tumor microenvironment and its overall interaction to provide much wider insight into the progression of cancer and response to treatment [185]. Such models are promising for the development of mathematical models of cancer mechanisms and treatment (Figure 1). Another advantage of representing cancer mechanisms in terms of mathematical models is that it enables easy design and implementation of algorithms for optimizing drug dose and effective treatment schedules [35,186]. Even though many such optimization results are reported for various cancers [31,158], only a few studies are reported specifically for BC [36]. Even though many such optimization results are reported for various cancers [31,35,158,186], only a few studies are reported specifically for BC [36]. 

In short, more in vitro/in vivo experiments and clinical trials are imperative to understand the crosstalk between HER2 and PD-1/PD-L1 pathways and related tumor dynamics under monotherapy or combination therapy [1]. Experimental data already available in the literature pertaining to specific cancers can also be utilized to develop disease-specific mathematical models. In order to clarify how biological mechanisms and parameters discussed in Section 2 and Section 3 can be accounted for in a mathematical model, we suggest a general one by summarizing the models given in [1,54,55,71,145]. Specifically, we provide a general model to illustrate the complex and nonlinear dynamics involved in tumor growth and treatment-induced tumor regression, pertaining to HER2 and PD-1/PD-L1 pathways as:

(1) $$\frac{dT\left(t\right)}{dt}=C_{T}\left(r,k,T\left(t\right)\right)-S_{T}\left(d,\delta ,U_{50},A_{b},A_{f},x,T\left(t\right),E\left(t\right),U\left(t\right)\right),\;T\left(0\right)=T_{0},$$

(2) $$\frac{dE\left(t\right)}{dt}=C_{E}\left(s,\alpha ,g,T\left(t\right),E\left(t\right),U\left(t\right)\right)-S_{E}\left(e,a,\beta ,T\left(t\right),E\left(t\right),U\left(t\right)\right),\;E\left(0\right)=E_{0},$$

(3) $$\frac{dV\left(t\right)}{dt}=C_{V}\left(v,T\left(t\right),E\left(t\right),V\left(t\right)\right)-S_{V}\left(w,T\left(t\right),E\left(t\right),V\left(t\right)\right),\;V\left(0\right)=V_{0},$$

(4) $$\frac{dU\left(t\right)}{dt}=C_{U}\left(u\left(t\right),V\left(t\right)\right)-S_{U}\left(\delta ,U\left(t\right)\right),\;U\left(0\right)=U_{0},$$

where T(t),E(t),V(t), and U(t) represent tumor cells, immune (effector) cells, vascular delivery in tumor, and drug concentration, respectively, $C_{T}\left(r,k,T\left(t\right)\right)$ is a function that accounts for contributing factors in tumor growth, where the parameters r and k are related to growth rate and carrying capacity, $S_{T}\left(d,\delta ,U_{50},A_{b},A_{f},x,T\left(t\right),E\left(t\right),U\left(t\right)\right)$ is a function that accounts for suppressing factors in tumor growth, where the parameters d,δ and $U_{50}$ are drug effect, drug decay rate (half-life/elimination), and drug concentration that causes 50% drug effect, respectively. 

In the context of HER2-targeted treatment and/or immune checkpoint inhibition which involve the use of antibodies and inhibitors, the parameter $A_{b}$ accounts for antibody binding, internalization of neoplastic drug, or target association of inhibitor; similarly, $A_{f}$ models free antibody, free neoplastic drug, and the parameter x denotes level of HER2 or PD-1/PD-L1 expressions. For instance, as mentioned in Section 2, the number of payloads of an antibody–drug conjugate or whether the antibody binds into the internal or extracellular domain of the HER2 receptor can influence the value of the parameters $A_{b}$ and $A_{f}$. Similarly, the function $C_{E}\left(s,\alpha ,g,T\left(t\right),E\left(t\right),U\left(t\right)\right)$ in Equation (2) denotes contributing factors related to effector cells, where s represents influx rate (or growth rate) of effector cells to the tumor microenvironment, α denotes activation rate (quiescent state to hunting state) of effector cells, and g represents immune-boosting facilitated by an immunotherapeutic drug. Likewise, the function $S_{E}\left(e,a,\beta ,T\left(t\right),E\left(t\right),U\left(t\right)\right)$ denotes suppressing factors related to effector cells, where e denotes a parameter that quantifies immune escape/evasion, a denotes apoptosis rate of effector cells, and β denotes the rate of inactivation of immune cells upon encounter with tumor cells. The functions $C_{V}\left(v,T\left(t\right),E\left(t\right),V\left(t\right)\right)$ and $S_{V}\left(w,T\left(t\right),E\left(t\right),V\left(t\right)\right)$ model the increase and decrease in vascular delivery, respectively, where v and w denote the rate of increase and decrease, respectively, with respect to tumor volume and immune response. Finally, $C_{U}\left(u\left(t\right),V\left(t\right)\right)$ and $S_{U}\left(\delta ,U\left(t\right)\right)$ model factors that affect drug concentration in terms of drug infusion rate u(t) and drug decay δ.

All of the eight functions given in Equations (1)–(4) can be modeled in different forms. For instance, tumor growth with respect to growth rate and carrying capacity can be modeled as rT(t), $e^{-rt}T\left(t\right),r\left(1-\frac{T\left(t\right)}{k}\right)T\left(t\right)$, or $rln\left(1-\frac{k}{T\left(t\right)}\right)T\left(t\right)$ which represent linear, exponential, logistic, or Gompertz equations, respectively. Similarly, drug effect terms can take a linear or exponential form. The Michaelis Menten function, sigmoid function, and Holling’s type functions are other common nonlinear forms used to account for the saturation effect involved in drug dynamics. Apart from the factors considered in Equations (1)–(4), dynamics related to hypoxia, necrosis, secretion of chemokines, etc., will also come into the picture according to the application for which a mathematical model is devised. In the case of immune checkpoint inhibition, effector cell dynamics (Equation 2) can be extended to model concentrations of IL-4, IL-12, IFN-γ, and GM-CSF, as well as density of dendritic cells and natural killer cells [71,145]. Moreover, with respect to the general model, parameters can be added or dropped according to their significance as determined by experimental studies.

The fact that many ongoing clinical trials are particularly dedicated to the development of mathematical models or to validate such models for BC (NCT03792529, NCT03983538, NCT02028494, NCT03381092) emphasizes the potential of theoretical analysis towards contributing to cancer care. Parallel to this, analysis is desirable to better understand cancer mechanisms and to improve the treatment of HER2^+^ BC via the use of immune checkpoint pathways along with anti-HER2 drugs. Specifically, biomarkers discussed in Section 2 and Section 3 related to drug resistance development, PD-L1 expression, prognosis of HER2+ BC, and treatment response can be considered while developing associated mathematical models. In essence, we list some of the opportunities on how mathematical models can contribute to improve the therapy of HER2^+^ BC as;

- It is imperative to develop mathematical models related to drug resistance development pertaining to HER2^+^ BC. Such models can be used to forecast the long-term efficacy of novel drugs, their combinations, or hypothetical treatment strategies [87,89].
- Out of the 5 FDA-approved anti-HER2 drugs, only the dynamics of trastuzumab and T-DM1 have been studied using a mathematical model to a certain extent. The dynamics of other drugs have yet to be explored on a quantitative basis. Such drug-specific models can be used for treatment planning and dose optimization [30,34,35,64,160,187].
- Developing mathematical models in terms of the biomarkers related to disease prognosis (e.g., PDL1 expression + high Tregs + less TILs = poor prognosis), and treatment response (e.g., presence of TILs favors response to trastuzumab) can help to identify patient cohorts that will benefit from a certain therapy [146].
- Mathematical models can be used to quantify drug dynamics of potential new drugs and different combinations and to explore possible additive or synergistic drug interaction when used in combinations [52].

## 5. Conclusions and Future Perspectives

Based on the significance of the PD-1/PD-L1 axis during carcinogenesis, including that in the HER2^+^ subtype of BC, it is crucial to elucidate the exact mechanism behind the interaction between the PD-1/PD-L1 pathway and HER2. This is essential to the discovery of new therapies as well as protocols for the management of HER2^+^ BC. On the other hand, we believe that developing new in vitro and in vivo experimental models is required for a better understanding of cancer mechanisms and the development of mathematical models. Additionally, since simulating clinical situations using mathematical models to evaluate the effect of various biological aspects of cancer management is cost-effective and safe, more collaborative efforts that take advantage of such mathematical models are essential to improve the management of cancer diseases.
